# Micrographia in Parkinson's Disease: Automatic Recognition through Artificial Intelligence

**DOI:** 10.1002/mdc3.70208

**Published:** 2025-07-07

**Authors:** Francesco Asci, Gaetano Saurio, Giulia Pinola, Marco Falletti, Alessandro Zampogna, Martina Patera, Francesco Fattapposta, Simone Scardapane, Antonio Suppa

**Affiliations:** ^1^ Department of Neurosciences and Sensory Organs AO San Giovanni—Addolorata Rome Italy; ^2^ Department of Computer, Control and Management Engineering Sapienza University of Rome Rome Italy; ^3^ Department of Human Neurosciences Sapienza University of Rome Rome Italy; ^4^ IRCCS Neuromed Institute Pozzilli Italy; ^5^ Department of Information Engineering, Electronics and Telecommunications Sapienza University of Rome Rome Italy

**Keywords:** artificial intelligence, handwriting, machine learning, Parkinson's disease, telemedicine

## Abstract

**Background:**

Parkinson's disease (PD) leads to handwriting abnormalities primarily characterized by micrographia. Whether micrographia manifests early in PD, worsens throughout the disease, and lastly responds to L‐Dopa is still under scientific debate.

**Objectives:**

We investigated the onset, progression and L‐Dopa responsiveness of micrographia in PD, by applying a non‐invasive and cheap tool of artificial intelligence‐ (AI)‐based pen‐and‐paper handwriting analysis.

**Methods:**

Fifty‐seven PD undergoing chronic L‐Dopa treatment were enrolled, including 30 early‐stage (H&Y ≤ 2) and 27 mid‐advanced stage (H&Y > 2) patients, alongside 25 age‐ and sex‐matched controls. Participants completed two standardized pen‐and‐paper handwriting tasks in an ecological scenario. Handwriting samples were examined through clinically‐based (ie, perceptual) and AI‐based (ie, automatic) procedures. Both consistent (ie, average stroke size) and progressive (ie, sequential changes in stroke size) micrographia were evaluated. Receiver operating characteristic (ROC) curves were used to evaluate the accuracy of the convolutional neural network (CNN) in classifying handwriting in PD and controls.

**Results:**

Clinically‐ and AI‐based analysis revealed a general reduction in stroke size in PD supporting the concept of parkinsonian micrographia. Compared with perceptual analysis, AI‐based analysis clarified that micrographia manifests early during the disease, progressively worsens and poorly responds to L‐Dopa. The AI models achieved high accuracy in distinguishing PD patients from controls (91%), and moderate accuracy in differentiating early from mid‐advanced PD (77%). Lastly, the AI model was not able to detect patients in OFF and ON states.

**Conclusions:**

AI‐based handwriting analysis is a valuable non‐invasive and cheap tool for detecting and quantifying micrographia in PD, for telemedicine purposes.

Micrographia is a well‐recognized clinical motor sign in Parkinson's disease (PD), referring to an overall decrease in stroke size and movement speed (ie, *consistent micrographia*) associated with a progressive decrement in stroke size of handwriting recordings (ie, *progressive micrographia*).^1^ Epidemiologic studies have reported micrographia in 10–15% of overall cohorts of PD,^2^ and more recent investigations found an even higher prevalence reaching about 70%.^3^

Several clinical and experimental studies have examined handwriting recordings in PD and reported specific features associated with consistent and progressive micrographia.^2^, ^3^, ^4^, ^5^, ^6^, ^7^, ^8^, ^9^ Moreover, kinematic analysis has deepened the understanding of micrographia in PD by detecting reduced stroke size and length, decreased stroke velocity and acceleration, and finally reduced pen pressure and fluency.^10^, ^11^, ^12^ However, owing to the biological complexity of handwriting tasks, innovative technological strategies are warranted to better examine micrographia in PD. Accordingly, applying artificial intelligence (AI) for automatically detecting handwriting abnormalities in PD would reasonably provide a novel and helpful solution for the objective detection of parkinsonian micrographia.^13^

So far, none has used non‐invasive and cheap AI‐based handwriting analysis to examine pen‐and‐paper samples for detecting consistent and progressive micrographia, automatically, in patients with PD. Also, although previous studies have demonstrated that micrographia may manifest early in PD, even before the clinical diagnosis,^2^, ^12^, ^14^ no studies have specifically used AI‐based procedures to achieve automatic recognition of micrographia, through objective handwriting analysis, in the early stage of the disease. Furthermore, none has clarified whether micrographia progressively deteriorates with disease progression nor verified the objective impact of L‐Dopa on handwriting tasks in PD. Lastly, none has applied a non‐invasive and cheap AI‐based pen‐and‐paper tool of handwriting analysis to detect micrographia, for identifying pathophysiological underpinnings of handwriting abnormalities and for telemedicine purposes, in an ecological scenario. Filling all these knowledge gaps will increase the current understanding of clinical features of micrographia in PD and promote the future application of AI‐based handwriting analysis in a telemedicine scenario.

We here examined and compared handwriting samples consisting of two standardized tasks, collected from a cohort of PD patients and age‐ and sex‐matched healthy subjects (HS), in a real‐life setting. We first perceptually inspected handwriting samples using clinically based (ie, perceptual) procedures. Then, to achieve objective measures of consistent and progressive micrographia, we digitalized and extracted several features including the overall pen stroke area and the progressive reduction in stroke size. To clarify the effect of the disease on handwriting samples, we used a convolutional neural network (CNN) model applied to pen‐and‐paper samples of handwriting recordings, to automatically discriminate PD samples from those recorded in HS. The CNN model was used to monitor disease progression by comparing handwriting samples recorded in cohorts in different stages of the disease (ie, early‐stage and mid‐advanced stage). Furthermore, to verify the impact of dopaminergic replacement treatment on handwriting, we compared recordings from OFF versus ON states. The diagnostic performances were assessed in detail for all comparisons (ie, sensitivity, specificity, positive and negative predictive values and accuracy). We also calculated the area under the receiver operating characteristic (ROC) curves to verify the optimal diagnostic threshold as reflected by the associated criterion (Ass. Crit.) and Youden Index (YI). Lastly, to assess possible correlations between patients’ clinical features and AI‐based measures of micrographia, we calculated the likelihood ratios (LRs) using the predicted outcome probabilities from the CNN model.

## Methods

### Participants

A 57 patients with PD (23 females; mean age: 68.9 ± 7.9 years) and 25 age‐matched HS (14 females; mean age: 67.4 ± 8.3 years) were enrolled in the study. Participants were recruited from the movement disorder outpatient clinics, at the Department of Human Neurosciences, Sapienza University of Rome, and IRCCS Neuromed Institute, Italy. Participants gave written informed consent, which was approved by the institutional ethics committee according to the Declaration of Helsinki. All subjects were native Italian speakers and right‐handed according to the Edinburgh Handedness Inventory.^15^ PD was diagnosed according to current criteria.^16^, ^17^ No disorders affecting handwriting, including orthopedic and rheumatologic diseases were reported by participants. The stage of the disease was assessed through the Hoehn & Yahr (H&Y) scale whereas clinical symptoms were scored using the Unified Parkinson's Disease Rating Scale part‐III (UPDRS‐III).^18^ Cognitive functions were assessed using the Mini‐Mental State Evaluation (MMSE)^19^ and the Frontal Assessment Battery (FAB),^20^ whereas mood was evaluated using the Beck Depression Inventory (BDI).^21^

The overall PD cohort included 30 early‐stage patients (H&Y ≤ 2) (15 females; mean age: 68.4 ± 8.7 years), and 27 mid‐advanced stage patients (H&Y > 2) (8 females; mean age: 69.3 ± 6.8 years). All patients were chronically treated with L‐Dopa and other dopaminergic agonists to improve their motor and non‐motor symptoms. Patients were studied at least 12 hours after the last L‐Dopa intake (ie, OFF state) and 1–2 h after the intake of the first dose of L‐Dopa (ie, ON state), randomly. Participant demographic and clinical features are summarized in Table 1.

**TABLE 1:** Demographic and clinical features of healthy subjects and PD

	Number (F:M)	Age (years)	Weight (kg)	Height (cm)	DD (years)	Mini‐mental state evaluation	Frontal assessment battery	H&Y	LEDDs	UPDRS‐III OFF	UPDRS‐III ON
PD (whole group)	57 (23:34)	68.9 ± 7.9	71.7 ± 11.8	168.6 ± 7.6	7.3 ± 5.6	28.1 ± 1.6	16.6 ± 1.3	2.0 ± 0.8	582.4 ± 275.3	25.6 ± 12.7	20.6 ± 11.0
*Early‐stage* PD	30 (15:15)	68.4 ± 8.7	71.0 ± 12.7	168.2 ± 8.4	4.8 ± 3.4	28.7 ± 1.6	16.7 ± 1.4	1.4 ± 0.5	429.2 ± 195.3	18.3 ± 6.2	14.2 ± 5.5
*Mid‐advanced‐stage* PD	27 (8:19)	69.3 ± 6.8	72.4 ± 11.0	169.1 ± 6.8	10.1 ± 6.3	27.6 ± 1.3	16.4 ± 1.2	2.8 ± 0.4	752.6 ± 252.1	33.7 ± 13.2	27.7 ± 11.4
Healthy subjects	25 (14:11)	67.4 ± 8.3	72.8 ± 8.8	172.6 ± 10.7	‐	29.0 ± 1.5	17.5 ± 1.2	‐	‐	‐	‐

*Note*: Results are expressed as average ± standard deviation.

Abbreviations: DD, disease duration; H&Y, Hoehn and Yahr Scale; LEDDs, L‐Dopa equivalent daily doses; OFF, not‐under L‐Dopa; ON, under L‐Dopa; PD, patients with Parkinson's disease; UPDRS‐III, Unified Parkinson's Disease Rating Scale part III.

### Handwriting Task

A written protocol with instructions for completing the handwriting tasks was sent to participants by the authors using the institutional email address. Following the enrollment, all subjects received a preliminary supervised training trial to familiarize themselves with the experimental procedures. Then, participants were asked to perform the handwriting tasks while sitting on a chair, with their arms lying on a table, at home and in the morning. The handwriting tasks consisted of writing: (1) the participant's given and family name and: (2) a semantic‐balanced Italian sentence “Questa è la mia scrittura”—“This is my handwriting.” These specific tasks were selected to avoid cultural factors, and the contribution of higher cognitive abilities related to the symbolic aspects of handwriting.^22^ Participants were preliminary provided with a fold of white A4 (210 × 297 mm) paper sheet (Fabriano, PG, Italy), and a couple of black ballpoint pen types (Bic, Clichy, France). Participants were asked to complete the handwriting tasks 10 times, consecutively, starting from the upper left side of the paper sheet and then proceeding downward, in a single column, with a self‐paced and comfortable speed. After collecting the handwriting task, participants were asked to digitalize the written paper sheet using the camera included in their smartphone (required resolution of at least five Megapixels). After scanning the handwriting samples, participants were asked to convert photos into PDF files using dedicated apps. Finally, participants completed the requested procedures by sending the PDF files to the authors’ institutional email server, and the files were stored anonymously on a dedicated Drive, encrypted, and password protected.

### Clinically‐Based Handwriting Analysis

The perceptual analysis was performed by a neurologist expert in movement disorders and blinded to the study, by using a modified visual analogue scale (VAS). The second handwriting tasks (ie, semantic‐balanced Italian sentences) were scored in HS and the overall cohort of patients OFF and ON state, by attributing an ordinal semi‐quantitative value ranging from 0 to 10, to the following handwriting features: the average height, the average length and the progressive size variation of both parameters. More in detail, 0 consisted of the smallest height and length and the greatest progressive reduction of previous parameters and 10 referred to the highest height and length as well as the greatest progressive increase of both variables. The VAS score for each participant was calculated by averaging the three previous values. To objectively assess consistent and progressive micrographia, pen stroke areas were calculated in a subset of 19 HS and 15 PD patients OFF/ON state (7 early‐ and 8 mid‐advanced stage). The pen stroke area of the first row in the handwriting task (ie, semantic‐balanced Italian sentence) was calculated for assessing consistent micrographia. Then, the ratio between averaged pen stroke areas of the first and last five rows of the handwriting task was calculated to examine progressive micrographia.

### AI‐Based Handwriting Analysis

AI‐based handwriting analysis was performed according to standardized procedures available online following previous studies in the field,^23^, ^24^, ^25^ including those based on deep learning algorithms.^26^, ^27^ In sum, the dataset of handwriting samples (ie, PDF files) was increased through a pre‐augmentation process consisting of manually cropping and extracting each of the 10 instances from each scanned paper sheet on a row basis, for consistency. After collecting PNGs, the dataset of handwriting samples was ensembled into specific and individual classes: (1) HS and (2) the overall cohort of PD in the OFF state. Then, four further subsets were generated: (1) early‐ and (2) mid‐advanced stage patients as well as: (1) OFF and (2) ON state. The preprocessing procedures allowed the collection of 1400 observations per class for the first (HS vs. PD) and third (OFF vs. ON) comparison and 700 observations for the second comparison (early vs. mid‐advanced stage). The classification analysis consisted of standard deep‐learning techniques, which were selected for their robust findings and translation invariance, allowing the extraction of hierarchical features.^28^, ^29^, ^30^ In particular, we fine‐tuned several models pre‐trained on the ImageNet dataset on the collected dataset.^31^ We adopted EfficientNet^32^ as architecture for the backbone, and fine‐tuned a small additional linear layer on top of the extracted features (see Supplementary Material S1). Multiple configurations for hyperparameters (concerning the choice of base model, the size of the classification layer, and the optimization process) were tested in training the model to improve the neural network generalization capability.

**FIG. 1:**
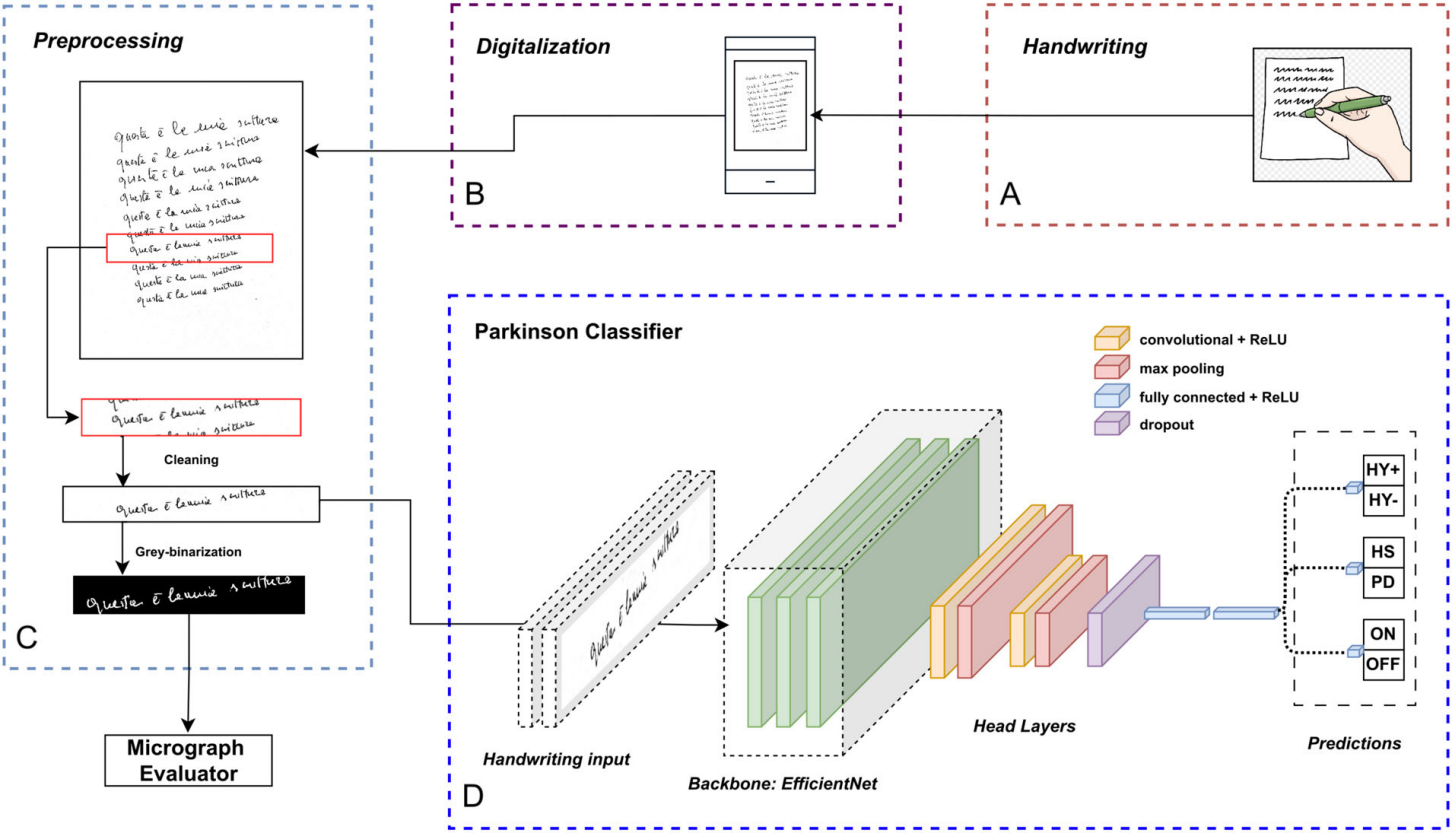
Experimental procedures and artificial intelligence (AI)‐based analysis: (A) Acquisition of handwriting samples; (B) Digitalization of the handwriting samples; (C) Preprocessing of the handwriting samples; (D) Model of deep learning architecture.

Moreover, to evaluate the model performance and obtain robust predictions, we employed a 5‐fold cross‐validation approach, where each fold was composed of all instances belonging to approximately one fifth of the original patients, for reducing potential overfitting. Specifically, we trained five versions of our neural network on different subsets of the data and tested them on corresponding validation sets. For each patient, we calculated the mean predicted probability across all folds, which we then used as the basis for determining the patient‐specific LR, ranging from 0 to 1 and reflecting the degree of handwriting impairment in each HS and PD patient (ie, the closer the LR to 1, the higher the degree of handwriting impairment). AI‐based procedures are reported in detail in Figure 1 and Supplementary Material S1, including a table showing the hyper‐parameters for the fine‐tuning of the classification model and a figure depicting the accuracy and loss plot, averaged over the folds, for the training and validation sets.

### Statistical Analysis

The normal distribution of demographic (ie, age) and anthropometric (ie, weight and height) features of HS and PD, including early‐stage and mid‐advanced stage patients, was assessed using the Kolmogorov–Smirnov test. The Chi‐Square Test was used to compare gender in HS and PD. The Mann–Whitney *U* test was used to compare demographic and anthropometric parameters in HS and PD, as well as clinical scores (ie, H&Y, UPDRS‐III, MMSE and the FAB) in early‐stage and mid‐advanced stage PD. The Wilcoxon signed‐rank test was used to compare UPDRS‐III scores in PD OFF/ON states. The unpaired Student *t*‐test was used to compare VAS scores in HS and PD and in early‐ and mid‐advanced‐stage patients; paired Student *t*‐test was used to compare the same measures in OFF/ON state. Likewise, we used the unpaired Student *t*‐test to compare the “pen stroke area” and the “size effect” in a subset of 19 HS and 15 PD patients. ROC analyses were calculated to identify the optimal diagnostic cut‐off values to discriminate between HS and PD, early‐stage and mid‐advanced stage patients, and finally patients OFF/ON state. For each ROC curve, we analyzed the YI and its associated criterion (ie, optimal threshold), as well as the Sensitivity, Specificity, Positive Predictive Value, Negative Predictive Value, Accuracy and Area Under the Curve. Spearman's rank correlation coefficient was used to assess correlations between clinical scores and LR values.

A *p*‐value <0.05 was considered statistically significant. Statistical analyses were conducted using STATA v17.0 (StataCorp LLC, USA).

## Results

Demographic and anthropometric parameters were normally distributed in HS and PD, including early‐stage and mid‐advanced‐stage patients (all *p* > 0.05). The Chi‐square test showed a balanced distribution of gender in HS and PD, and lastly in early‐stage and mid‐advanced stage patients (all *p* > 0.05). Age, weight, height, and BMI were all comparable among groups (all *p* > 0.05). MMSE and FAB scores were lower, whereas BDI scores were higher in PD compared to HS (all *p* < 0.05). Cognitive and mood assessments were comparable between early‐stage and mid‐advanced stage patients (all *p* > 0.05).

As expected, the disease duration and LEDDs were lower in early‐stage than in mid‐advanced stage patients (all *p* < 0.01). Also, mid‐advanced stage showed higher scores at UPDRS‐III than early‐stage patients (*p* < 0.05). Concerning the effect of L‐Dopa, as expected we found lower UPDRS‐III scores in ON than OFF state (OFF:25.6 ± 12.7; ON:20.6 ± 11.0; *z* = −6.50; W = 0; *p* < 0.05). A comparable symptomatic effect following L‐Dopa administration was observed in early‐stage (OFF:18.3 ± 6.2; ON:14.2 ± 5.5; *z* = −4.78; W = 0; *p* < 0.05) and in mid‐advanced stage patients (OFF:33.7 ± 13.2; ON:27.7 ± 11.4; *z* = −4.46; W = 0; *p* < 0.01) (Table 1).

### Clinically‐Based Handwriting Analysis

Less than 5% of samples collected in HS and PD were discarded after a preliminary perceptual analysis when judged of poor quality. All clinically‐based measures are reported in Table 2.

**TABLE 2:** Micropraphia assessment in healthy subjects and PD

	Healthy subjects	PD OFF	PD ON	Early‐stage PD OFF	Early‐stage PD ON	Mid‐advanced‐stage PD OFF	Mid‐advanced‐stage PD ON
VAS score	5.93 ± 1.15	3.91 ± 0.99	4.12 ± 0.97	4.17 ± 1.17	4.13 ± 1.09	3.79 ± 0.91	4.10 ± 0.83
Pen stroke area	126.72 ± 44.85 mm^2^	89.78 ± 26.37 mm^2^	93.82 ± 31.20 mm^2^	84.81 ± 27.99 mm^2^	84.34 ± 23.84 mm^2^	94.14 ± 25.94 mm^2^	102.11 ± 35.93 mm^2^
Sequence effect	1.05 ± 0.16	0.92 ± 0.23					

*Note*: Results are expressed as average ± standard deviation.

Abbreviations: OFF, not under L‐Dopa; ON, under L‐Dopa; PD, Parkinson's disease.

When comparing VAS scores collected in HS and PD, we found lower values in patients than controls, considering both OFF (*t* = 8.06; *p* < 0.01) and ON state (*t* = 7.39; *p* < 0.01). Conversely, the unpaired Student *t*‐test reported comparable results when comparing VAS scores collected in OFF/ON state patients and when comparing early‐ and mid‐advanced stage PD, when OFF/ON states (Table 2).

When comparing “pen stroke area,” we found smaller stroke areas in patients than controls, when OFF/ON state (OFF: *t* = 2.82; *p* < 0.01; ON: *t* = 2.41; *p* < 0.05). Moreover, the unpaired Student *t*‐test also disclosed progressive micrographia in PD but not in HS (*t* = 2.66; *p* < 0.05) (Table 2).

When focusing on the effect of disease progression and L‐Dopa, the unpaired Student *t*‐test reported comparable “pen stroke area” in early‐ and mid‐advanced stage (*t* = 0.67; *p* = 0.51), and in patients OFF/ON states (*t* = 0.80; *p* = 0.43) (Table 2).

### AI‐Based Handwriting Analysis

**FIG. 2:**
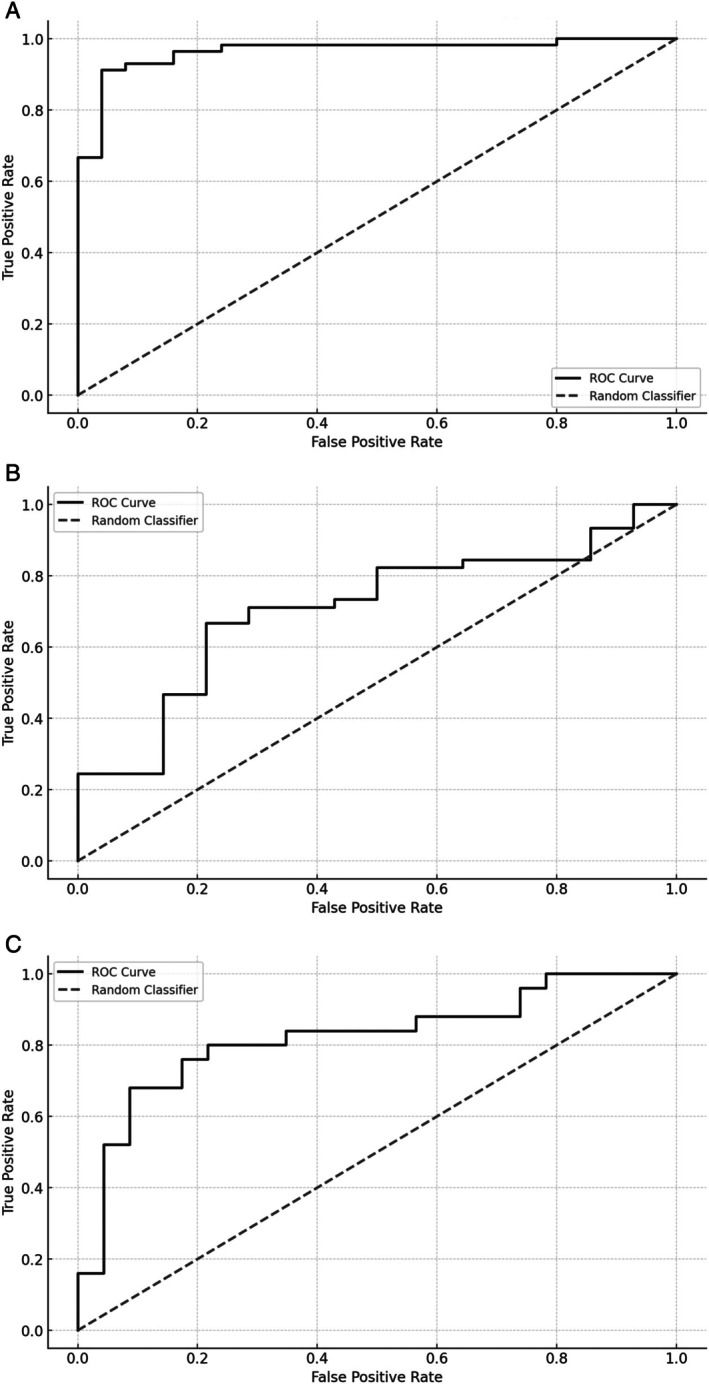
Convolutional Neural Network analysis. Receiver operating characteristic (ROC) curves were calculated to differentiate healthy subjects and patients with PD (A), patients in early‐ and mid‐advanced stages (B), patients in OFF and ON states (C).

When applying the CNN classifier to handwriting samples collected in HS and the overall cohort of PD, the ROC curve disclosed highly significant performances for both handwriting tasks. More in detail, the ROC curve analyses identified the following values of statistical significance: sensitivity = 93%, specificity = 88%, Positive Predictive Value = 95%, Negative Predictive Value = 85%, Accuracy = 91%, Area Under the Curve = 0.96 and YI = 0.81 (Fig. 2A).

When we compared handwriting samples in early‐ and mid‐advanced stage PD, the CNN classifier achieved a moderate performance when analyzing samples of both handwriting tasks. More in detail, the ROC curve analysis reported the following values of statistical significance: sensitivity = 72%, specificity = 83%, Positive Predictive Value = 82%, Negative Predictive Value = 73%, Accuracy = 77%, Area Under the Curve = 0.82 and YI = 0.55 (Fig. 2B).

Lastly, when applying the CNN for discriminating patients in OFF/ON states, we achieved a rather poor accuracy and the ROC curve analysis showed sensitivity = 56%, specificity = 79%, Positive Predictive Value = 89%, Negative Predictive Value = 35%, Accuracy = 61%, Area Under the Curve = 0.71 and YI = 0.35 (Fig. 2C).

### Clinical‐Instrumental Correlations

The second subitem of the VAS (ie, the average length of strokes) negatively correlated with the “pen stroke area” (*r* = −0.56, *p* < 0.05), an objective measure of micrographia. Also, the third subitem of our VAS (ie, the progressive size variation of average height and length of strokes) negatively correlated with UPDRS‐III OFF (*r* = −0.65, *p* < 0.01) and UPDRS‐III ON (*r* = −0.60, *p* < 0.05), that is, the higher the disease severity, the smaller the stroke size throughout handwriting as scored with perceptual scale, in patients OFF and ON state. Lastly, we found a negative correlation between LRs and progressive micrographia calculated with the AI‐based method (*r* = −0.66, *p* < 0.01), that is, the smaller the stroke sizes, the greater the degree of handwriting impairment in PD patients.

## Discussion

In this study using artificial intelligence, we demonstrated micrographia in PD by using clinically‐based and AI‐based analysis. Besides perceptual measures, the AI‐based procedures clarified that handwriting deteriorates early in PD, worsens along with disease progression, and remains unchanged following L‐Dopa administration. AI‐based handwriting analysis should be considered a safe, cheap, and eco‐friendly tool for detecting handwriting abnormalities in PD, in a culture‐free and ecological scenario, thus supporting its application for telemedicine purposes.

### Micrographia in Parkinson's Disease

Using our clinically‐based analysis, we demonstrated that handwriting samples in PD exhibit significantly diminished stroke sizes (consistent micrographia) and a progressive reduction in stroke size (progressive micrographia) compared to control subjects. Additionally, we demonstrated that progressive micrographia becomes more pronounced as the disease severity increases (ie, UPDRS‐III scores). These findings point to micrographia as a cardinal feature of parkinsonian handwriting in line with previous observations.^2^, ^33^ Seminal studies based on visual inspection of free handwriting samples have identified specific handwriting features in PD, including reduced stroke length, erratic and inconsistent writing appearance, retouching, and tremors, all categorized under the term micrographia.^2^, ^5^, ^12^, ^34^, ^35^ However, these investigations have been affected by substantial inter‐rater variability, which can be attributed to the intrinsic perceptual nature of the analysis.

In addition to the clinically‐based analysis, we here employed the AI‐based analysis to assess both consistent and progressive micrographia in PD, through static evaluations of the pen stroke area in handwriting samples. We first objectively confirmed the presence of consistent micrographia in PD by demonstrating that patients’ handwriting recordings are characterized by significantly smaller stroke size areas compared to controls.^1^, ^2^ Our findings are fully in line with those identified by previous seminal papers in the field based on AI algorithms. Comparable high values of accuracy in discriminating HS versus PD have been identified when using specific AI algorithms, including the K‐nearest neighbors (K‐NN), AdaBoost classifier, and support vector machines (SVM) to kinematic and pressure features collected during several handwriting tasks through tablet.^36^ Also, by using offline analysis based on pre‐trained multiple‐fine‐tuned CNNs to extract features from images capturing handwriting samples, the authors obtained high accuracies when using specific tasks.^37^, ^38^, ^39^, ^40^, ^41^, ^42^, ^43^ Moreover, high classification accuracies have been achieved by applying a blended methodology based on combined conventional handcrafted features and features extracted automatically by a pretrained CNN.^44^ Hence, both the clinically‐based and AI‐based procedures were able to recognize micrographia in PD as also confirmed by the correlation achieved between our perceptual evaluation of the average length of strokes and the AI‐based measures of “pen stroke area.”

In this study, we also provide the first automatic and objective measure of progressive micrographia in PD, defined as the gradual reduction in letter size from the beginning to the end of the writing sample.^1^, ^2^ Moreover, the greater the progressive micrographia the higher the accuracy in the automatic PD recognition (ie, LR values). These findings overall further corroborate prior observations indicating that progressive micrographia is a specific handwriting abnormality associated with PD.^2^, ^5^, ^12^, ^22^, ^34^, ^35^

### The Effect of Disease Progression Is Recognized by AI‐Based but Not Clinically‐Based Procedures

Our clinically‐based analysis failed to detect a worsening of micrographia along with disease progression, as demonstrated by comparable VAS scores in PD patients in the early‐ and mid‐advanced stages. Conversely, the AI‐based analysis unexpectedly distinguished handwriting samples recorded in patients in the early‐ and mid‐advanced stages supporting some previous kinematic observations.^41^, ^45^, ^46^, ^47^, ^48^ Our findings therefore point to the detrimental effect of disease progression on handwriting in patients with PD. The observation that the detrimental effect of disease progression on handwriting can be identified by AI‐based but not by clinically‐based procedures further confirm the need for more objective and automatic measures in the examination of handwriting in PD.

### The Effect of L‐Dopa: Converging Clinically‐ and AI‐Based Measures

Our clinically‐based analysis did not detect beneficial effects of L‐Dopa on micrographia in PD, as suggested by comparable VAS scores in patients OFF/ON states. Similarly, AI‐based analysis failed to identify differences in patients OFF/ON states. These findings overall point to the lack of responsiveness of PD handwriting abnormalities to L‐Dopa administration. Our observations only partly disagree with some investigations showing that L‐Dopa could improve simple but not complex writing tasks in PD.^4^, ^5^, ^7^, ^9^, ^31^, ^49^, ^50^ Previous studies have indeed reported a limited beneficial effect of L‐Dopa in specific sub‐items of handwriting, including consistent^6^ but not progressive micrographia.^7^, ^8^

### Pathophysiology of Micrographia in PD

The pathophysiology of parkinsonian micrographia is still largely unclear. Although previous studies have explored the potential contributions of peripheral nerve impairment,^51^, ^52^ muscle fibers alterations,^53^ and joint abnormalities,^54^ compelling evidence suggests that micrographia would reflect functional changes in the cortico‐basal ganglia‐thalamo‐cortical circuit.^6^, ^11^ Previous functional magnetic resonance imaging studies in patients with PD have demonstrated abnormal activity in the left (ie, dominant) posterior putamen, thalamus, and caudal supplementary motor area.^6^, ^11^, ^55^ Wu et al.^6^ showed that consistent micrographia correlated with decreased functional connectivity between Exner's area of the left premotor cortex and the posterior putamen, and increased connectivity between the left premotor cortex and anterior putamen. Besides consistent micrographia, the same study demonstrated that progressive micrographia reflects reduced connectivity of the left pre‐supplementary motor area, right rostral cingulate motor area, and cerebellum.^6^ We here speculate that parkinsonian micrographia would primarily result from functional changes in critical nodes of *the human writing network*.^56^ This neural network which is thought to underpin cognitive and motor processes involved in human writing, includes key cortical regions such as the angular gyrus and precentral gyrus (ie, lexical processes of handwriting), the left perisylvian regions (ie, phonological aspects), left superior parietal and premotor areas including Exner's area (ie, handwriting execution), alongside subcortical structures such as basal ganglia (ie, left putamen) and anterior cerebellum. Indeed, selective brain lesions in the angular gyrus/precentral gyrus/left perisylvian regions and in the left superior parietal/premotor regions are known to lead to dysorthographias (ie, lexical or phonological components) and apraxic agraphia, respectively.^55^, ^57^ Interestingly, structural lesions of the left putamen and thalamus may lead to micrographia.^58^ Overall, we speculate that micrographia in PD would reflect functional changes in dopaminergic (ie, putamen) as well as non‐dopaminergic structures (ie, premotor areas including Exner's area) included in the *human writing network*.^59^, ^60^ This hypothesis fits in well with previous clinical, neuroimaging and neuropsychological observations in patients with PD.^6^, ^7^, ^11^, ^55^

Several limitations should be considered when interpreting our findings. The number of patients included in our cohorts might be considered rather small. Accordingly, we cannot exclude that more ample cohorts would have allowed a better distinction between consistent and progressive micrographia concerning PD progression and response to L‐Dopa. Furthermore, we cannot exclude that intrinsic weaknesses of perceptual (ie, subjective) clinical assessment of handwriting would explain at least some of our results of a lack of effect of L‐Dopa on handwriting skills in PD. Moreover, it should be acknowledged, however, that consistent and progressive micrographia do coexist in varying degrees in patients with PD making it difficult a clear distinction among features.^11^, ^14^ Still, we are also aware that handwriting abnormalities in PD are not limited to micrographia since the spectrum of writing disturbances also includes dynamic (ie, abnormal force generation) and other kinematic features (duration, velocity, acceleration and fluency of writing).^11^, ^14^ Accordingly, besides micrographia, the term *PD dysgraphia* should be preferred when referring to all the handwriting changes limiting the ability of PD patients to perform fluent graphomotor movements.^11^, ^14^ Lastly, although our hypothesis that parkinsonian micrographia reflects functional changes in the human writing network is reasonable, we here did not provide evidence for a specific brain structure responsible for the pathophysiology of micrographia in PD.

Our study provides clinically‐ and AI‐based evidence of consistent and progressive micrographia in PD. Micrographia manifests early in PD, progressively worsens throughout the disease and poorly responds to L‐Dopa. Our findings highlight the multifaceted nature of micrographia in PD, emphasizing the role of *the human writing network* in the early onset and progression of both consistent and progressive micrographia. We believe that our findings would point to a significant role of artificial intelligence as a safe, cheap, and eco‐friendly tool for objectively assessing disease severity and progression in PD, for telemedicine purposes. The significant results obtained in this real‐world setting open several future research perspectives, such as exploring different algorithm setups and handwriting sample generation. In particular, we believe that collecting larger datasets over time, also through revised AI kinematic tools for better clinical assessment, would enable tracking disease progression using sequential (ie, recurrent) AI models trained on handwritten samples collected at regular intervals.

## Disclosure

**Ethical Compliance Statement:** The study was approved by the Ethics Committee of the Neuromed Institute in Pozzilli. Informed consent was obtained from all patients and documented in writing. We confirm that we have read the Journal's position on issues involved in ethical publication and affirm that this work is consistent with those guidelines.

**Funding Sources and Conflict of Interest**: No specific funding was received for this work. The authors declare that there are no conflicts of interest relevant to this work.

**Financial Disclosures for the Previous 12 Months:** The authors declare that there are no additional disclosures to report.

## Authors Roles

(1) Research project: A. Conception, B. Organization, C. Execution; (2) Statistical Analysis: A. Design, B. Execution, C. Review and Critique; (3) Manuscript Preparation: A. Writing of the first draft, B. Review and Critique.

F.A.: 1A, 1B, 1C, 2A, 2B, 3A.

G.S.: 1B, 1C, 2A, 2B.

G.P.: 1B, 1C, 2A, 2B, 3A.

M.F.: 1C.

A.Z.: 1C.

M.P.: 1C.

F.F.: 1C.

S.S.: 1B, 2A, 2C, 3A.

A.S.: 1A, 2A, 2C, 3A.

## Supporting information


**Data S1** Supporting Information.

## Data Availability

The data that support the findings of this study are available from the corresponding author upon reasonable request.
