# Supplementary material for: Micrographia in Parkinson's Disease: Automatic Recognition through Artificial Intelligence
Source: Mov Disord Clin Pract. 2025 Jul 7;12(12):2187–96. doi: 10.1002/mdc3.70208 (PMC12715321; doi:10.1002/mdc3.70208)
Supplement: Supplementary file 1 — Data S1 Supporting Information. [file MDC3-12-2187-s001.docx]

**Supplementary Material 1**

*Detailed report of the methodology applied for the artificial Intelligence (AI)-based handwriting analysis*

AI-based handwriting analysis was performed according to standardized procedures available online following previous studies in the field^1–3^, including those based on deep learning algorithms^4,5^. First, the dataset of handwriting samples (i.e., PDF files) was increased through a pre-augmentation process consisting of cropping and extracting each instance from each scanned paper sheet on a row basis. Then, the variability of scanning resolutions of PDF files was reduced through resizing and padding. As a result, cropped PNGs of 1000x166 containing well-centered and extended writing were extracted from the initial bounding boxes. After collecting the PNGs, the dataset of handwriting samples was ensembled into specific and individual classes to improve data acquisition for the following training process. More in detail, two subsets were extracted from the overall group of handwriting samples: 1) healthy subjects and 2) the overall cohort of PD OFF state. Then, four further subsets were generated: 1) early- and 2) mid-advanced-stage patients as well as: 1) OFF and 2) ON state. The number of instances in each subset was further increased using vertical and horizontal offline and online augmentation techniques. These preprocessing procedures allowed the collection of 1400 observations per class for the first (healthy subjects vs. PD) and third (OFF vs. ON) comparison and 700 observations for the second comparison (early vs. mid-advanced stage). A normalization technique based on the greyscale binarization function was used on input images to improve the background color of images, the color of strokes, signs or strokes. This process allowed the reduction of color channels and the conversion of the images into white-on-black text for uniformity.

The classification analysis consisted of fine-tuning a pre-trained neural network models, which was selected for its robust accuracy and translation invariance, allowing the extraction of hierarchical features^6–8^. In particular, according to standard practices, we fine-tuned several models pre-trained on the ImageNet dataset on the collected dataset^9^. We adopted EfficientNet^10^ as architecture for the backbone, testing multiple variants ranging from B0 to B7^10^. Also, various techniques and hyperparameter settings were analyzed in training the model to improve the neural network generalization capability. Ultimately, among all those tested and mentioned, the model that offered the best results in terms of generalization while maintaining greater simplicity was EfficientNet B0^10^. Details on the optimization procedure are provided in Supplemental Table 1. Moreover, to evaluate the model performance and obtain robust predictions, we employed a 5-fold cross-validation approach, where each fold was composed of approximately one-fifth of the overall cohort of patients (so that all instances of a single patient were used exclusively for either training or testing). After training five versions of our neural network on the training subsets, we tested them on the corresponding validation folds and, for each patient, we calculated the mean predicted probability across all folds, which we then used as the basis for determining the patient-specific likelihood ratios, ranging from 0 to 1 and reflecting the degree of handwriting impairment in each healthy subjects and PD patient (i.e., the closer the likelihood ratio to 1, the higher the degree of handwriting impairment). Lastly, PyTorch was used to implement the model and the pre-trained EfficientNet weights were extracted from the Hugging Face timm library. The convolutional neural network procedure is reported in Figure 1 in the main text, while the training and validation processes as loss and accuracy plots averaged over the folds have been shematically reported in the Supplemental Figure 1.

**REFERENCES**

1. Dey S, Dutta A, Toledo JI, et al. SigNet: Convolutional Siamese Network for Writer Independent Offline Signature Verification. Epub ahead of print 2017. DOI: 10.48550/ARXIV.1707.02131.

2. Hafemann LG, Sabourin R, Oliveira LS. Offline handwritten signature verification — Literature review. In: *2017 Seventh International Conference on Image Processing Theory, Tools and Applications (IPTA)*. Montreal, QC: IEEE, pp. 1–8.

3. Pang S, Yang X. Deep Convolutional Extreme Learning Machine and Its Application in Handwritten Digit Classification. *Computational Intelligence and Neuroscience* 2016; 2016: 1–10.

4. Liu Z, Jin L, Chen J, et al. A survey on applications of deep learning in microscopy image analysis. *Computers in Biology and Medicine* 2021; 134: 104523.

5. Islam MA, Hasan Majumder MZ, Hussein MA, et al. A review of machine learning and deep learning algorithms for Parkinson’s disease detection using handwriting and voice datasets. *Heliyon* 2024; 10: e25469.

6. LeCun Y, Boser B, Denker JS, et al. Backpropagation Applied to Handwritten Zip Code Recognition. *Neural Computation* 1989; 1: 541–551.

7. Bengio Y, Lecun Y, Hinton G. Deep learning for AI. *Commun ACM* 2021; 64: 58–65.

8. LeCun Y, Bengio Y, Hinton G. Deep learning. *Nature* 2015; 521: 436–444.

9. Kaur R, Kumar R, Gupta M. Review on Transfer Learning for Convolutional Neural Network. In: *2021 3rd International Conference on Advances in Computing, Communication Control and Networking (ICAC3N)*. Greater Noida, India: IEEE, pp. 922–926.

10. Le Q. TM. EfficientNet: Rethinking Model Scaling for Convolutional Neural Networks. In: *Chaudhuri K, Salakhutdinov R, editors. Proceedings of the 36th International Conference on Machine Learning*. PMLR, 2019, pp. 6105–14.

11. Loshchilov I, Hutter F. SGDR: Stochastic Gradient Descent with Warm Restarts. Epub ahead of print 3 May 2017. DOI: 10.48550/arXiv.1608.03983.

**Supplemental Table 1.** Hyper-parameters for the fine-tuning of the classification model

| Hyperparameter | Setup |
| --- | --- |
| Model | EfficientNet-B0^10^ – the final layer was replaced with a randomly-initilized binary classifier, and a dropout layer is added before the final layer to improve optimization. |
| Optimizer | Adam optimizer with learning rate 5e-4 |
| Learning rate scheduler | Cosine annealing^11^ with a weight decay of 1e-6 |
| Batch size | 32 (10 epochs in total) |

| 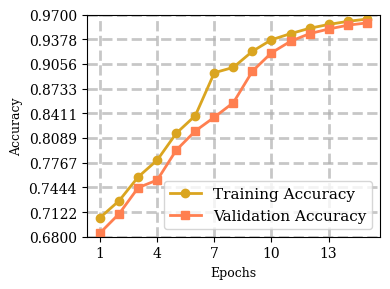   1. Accuracy | 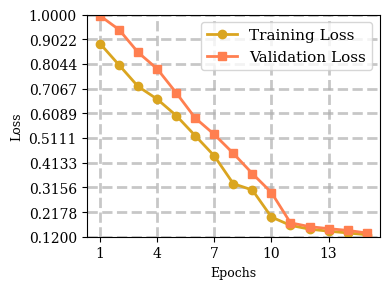   1. Loss |
| --- | --- |

**Supplemental Figure 1.** Accuracy and loss plot, averaged over the folds, for the training and validation sets
